# Huntingtin’s Function in Axonal Transport Is Conserved in *Drosophila melanogaster*

**DOI:** 10.1371/journal.pone.0060162

**Published:** 2013-03-28

**Authors:** Diana Zala, Maria-Victoria Hinckelmann, Frédéric Saudou

**Affiliations:** 1 Institut Curie, Orsay, France; 2 CNRS UMR 3306, Orsay, France; 3 Inserm U1005, Orsay, France; University of Massachusetts Medical School, United States of America

## Abstract

Huntington’s disease (HD) is a devastating dominantly inherited neurodegenerative disorder caused by an abnormal polyglutamine expansion in the N-terminal part of the huntingtin (HTT) protein. HTT is a large scaffold protein that interacts with more than a hundred proteins and is probably involved in several cellular functions. The mutation is dominant, and is thought to confer new and toxic functions to the protein. However, there is emerging evidence that the mutation also alters HTT’s normal functions. Therefore, HD models need to recapitulate this duality if they are to be relevant. *Drosophila melanogaster* is a useful *in vivo* model, widely used to study HD through the overexpression of full-length or N-terminal fragments of mutant human HTT. However, it is unclear whether *Drosophila* huntingtin (DmHTT) shares functions similar to the mammalian HTT. Here, we used various complementary approaches to analyze the function of DmHTT in fast axonal transport. We show that DmHTT interacts with the molecular motor dynein, associates with vesicles and co-sediments with microtubules. DmHTT co-localizes with Brain-derived neurotrophic factor (BDNF)-containing vesicles in rat cortical neurons and partially replaces mammalian HTT in a fast axonal transport assay. DmHTT-KO flies show a reduced fast axonal transport of synaptotagmin vesicles in motoneurons *in vivo*. These results suggest that the function of HTT in axonal transport is conserved between flies and mammals. Our study therefore validates *Drosophila melanogaster* as a model to study HTT function, and its dysfunction associated with HD.

## Introduction

The fruit fly *Drosophila melanogaster* (Dm) has various characteristics that make it a useful model for medical and fundamental research. For example, more than 75% of human disease genes have a homologue in flies [1], and the bipartite UAS-GAL4 system developed by Perrimon and collegues offers an extremely flexible tool to control, in space and time, the expression of transgenes [2]. Also, the numerous mutant and transgenic flies available, and the associated databases, are powerful tools for both large output screening and basic research.

For these reasons and despite obvious morphological, size and neuronal circuitry differences between human and fly brains, *Drosophila melanogaster* is widely used in neurodegenerative diseases research. In particular, several fly strains have been generated to model Huntington’s disease (HD), a dominantly inherited neurodegenerative disorder caused by an abnormal polyglutamine (polyQ) expansion in the huntingtin (HTT) protein [3]–[7]. These models are based on the overexpression of the full length, or N-terminal fragments of, human HTT. Flies expressing mutant HTT show neuronal dysfunction, such as defects in synaptic transmission and axonal transport, neuronal degeneration, locomotor deficits and shorter lifespans [4]–[8]. HD fly models have been extensively used to validate candidate approaches and to search for gene modifiers that rescue neurodegeneration [9]–[15]. Importantly, these studies are based on the notion that the mutation in HTT results in a gain of new toxic functions that are unrelated to wild-type HTT function. Indeed, the HD mutation is dominant and the overexpression of the HTT fragments containing the polyQ expansion is sufficient to induce phenotypes in flies. However, recent evidence in mammals suggests that alteration of the wild-type HTT functions also contributes to HD [16]–[19]. This duality, both gain and loss of function, of the pathogenic mechanisms raises the issues of whether the overexpression of polyQ HTT in flies faithfully recapitulates mammalian HD, and the degree to which HTT function is conserved between flies and mammals.

HTT is a large scaffold protein of 350 kDa in human and of a predicted 400 kDa in fly. HTT interacts with hundreds of proteins and regulates several cellular functions [14], [16], [18], [20], [21]. For example, laboratories have reported that HTT is a positive regulator of microtubule-(MT)-based transport [22]–[25]. This function is altered upon polyQ expansion [22], [24] and vesicular transport is slowed down as a result.

Three studies have investigated the function of *Drosophila* HTT (DmHTT) in axonal transport [24], [26], [27] but there are discrepancies between their findings. Silencing DmHTT by sh-RNA resulted in accumulation of axonal organelles, characteristic of severe transport defects [24]. This phenotype was more evident in kinesin heavy chain heterozygous flies. By contrast, the second study reported that HTT knock-out flies are viable with no obvious developmental defects and normal axonal transport [26]: no synaptotagmin accumulation was observed in axons. However, neither study directly assessed the dynamic nature of vesicles in axons through the observation of fluorescent cargo by videomicroscopy [28]. Importantly, in mammals, in contrast to the silencing or depletion of molecular motors, HTT silencing reduces but does not totally block axonal transport of cargo and does not result in the accumulation of axonal organelles. Finally, a recent study reported a defect in the dynamics of Rab11 but not Rab5 vesicles in *Drosophila* larvae in which DmHTT was silenced by RNAi [27]. These results suggest that DmHTT could play an important role in flies.

Here, we report the study, by various complementary approaches, of the role of HTT in fast axonal transport. Our results indicate that the function of HTT in axonal transport is evolutionarily conserved between flies and mammals.

## Materials and Methods

### Statistical Analyses

Statview 4.5 software (SAS Institute Inc.) was used for statistical analysis. Groups were compared by ANOVA followed by Fisher’s PLSD post hocs analyses. The criterion for statistical significance was set at *P*<0.05. Error bars, S.E.M.

### Flies

*D. melanogaster* flies were stocked at 18°C on a fly standard medium. Recombinant *ok6-Gal4:UAS-Syt-GFP* (X) flies were a kind gift from ML Parmentier, control w118 flies and DmHTT-Knock out flies are a kind gift from S. Zhang and N. Perrimon [26]. Flies were maintained at 25°C for the experiments. Offspring L3 larvae were collected for video-microscopy. 3–5 larvae from the same genotype were laid on an 18 mm round coverslip. A second coverslip was added to form a sandwich and the system was mounted in a modified homemade Ludin chamber with a gentle compression of the larvae. Larvae were then observed by fast-video-microscopy.

### Plasmids and Antibodies

Dm620HTT-GFP was previously described [29]; GFP was obtained from Invitrogen (pCDNA-N1-GFP). *Drosophila* antibody (DmHTT-S50) was generated by immunizing rabbits with two peptide sequences: H2N-RHRRDRNKAKGPAPQC-CONH2 and H2N-CVASDEDKQGQGHRQQ-CONH2 (Eurogentec, Rabbit DX Speedy-2 peptides program). Antisera analysis by indirect ELISA was performed to quantify the response of different bleeds. The following antibodies and dilutions were used for immunoblotting experiments: rabbit anti DmHTT (DmHTT-S50, 1∶1∶500) rabbit anti GFP (1∶1000, Institut Curie), mouse anti DIC (1∶2000, Millipore), mouse anti huntingtin (4C8, 1∶3000, Euromedex), mouse anti p150*^Glued^* (1∶1000, BD Transduction Laboratories), mouse anti α-tubulin (1∶3000, Sigma-Aldrich), mouse anti β-actin (1∶5000, Sigma-Aldrich).

### Immunoprecipitation Experiments

HEK 293 cells were transfected with GFP or Dm620HTT-GFP using calcium phosphate-based method. Two days post-transfection, cells were washed once with ice-cold PBS and pelleted. Cells were lyzed in the following buffer; 50 mM Tris pH8.0, 150 mM NaCl, 1% NP40, 1∶100, containing a cocktail of proteinase inhibitor (Sigma-Aldrich) and PMSF 1 mM (Sigma-Aldrich). Immunoprecipitation was performed with µMACS GFP-Tagged Protein Isolation Kit according to the provided protocol (MACS molecular). Anti-HA beads were used as negative controls (MACS molecular).

### Microtubule Depolymerization-Repolymerization Assay

HEK 293 cells where lyzed by passing the samples 10 times in a 15 G syringe in ice cold BRB80 buffer (Pipes 80 mM, MgCl_2_ 1 mM and EGTA 1 mM) containing a cocktail of protease inhibitor (Sigma-Aldrich) and PMSF 1 mM (Sigma-Aldrich). Samples were clarified at 10,000×*g* for 30 min at 4°C. Supernatant (Total) was incubated at 37°C with 1 mM GTP for 15 min, then Taxol 50 µM was added to stabilized microtubules for 15 min at 37°C. For negative control, Taxol was replaced by nocodazole 50 µM. Samples were centrifuged at 10,000×*g* for 30 min at 30°C and supernatant and pellet containing microtubules and associated proteins were collected. Pellet was re-suspended in one fifth of the supernatant volume.

### Subcellular Fractionation

HEK 293 cells were lyzed by passing the samples 10 times in a 15 G syringe in ice cold Hepes 4 mM and sucrose 320 mM buffer containing a cocktail of proteinase inhibitor (Sigma-Aldrich) and PMSF 1 mM (Sigma-Aldrich). Samples (Total) were centrifuged at 3000 *g* for 10 min at 4°C to recover pellet (P1) and supernatant (S1). S1 was centrifuged at 10,000×*g* for 30 min at 4°C to recover pellet (P2) and supernatant (S2). S2 was centrifuged at 90,000×*g* for 90 min at 4°C to recover pellet (P3) and supernatant (S3). P1 and P2 were re-suspended respectively in the same volumes of S1 and S2 samples. P3 was re-suspended in one fifth of the S3 volume.

### Neuronal Cultures

Primary striatal neurons were prepared from E17 rat embryos as previously described [30]. Briefly, cortices were dissected in cold DM buffer pH 7.4 (Na_2_SO_4_ 82 mM K_2_SO_4_ 30 mM, MgCl_2_ 5.8 mM, CaCl_2_ 250 nM, Hepes 1 mM, Glucose 20 mM and Phenol red) supplemented with kynurenic acid 1 mM and MgCl_2_ 8 nM. Tissue digestion was performed with 2 incubations of 10 min in a solution of papain and cysteine followed by 2 incubations of 7 min and 30 sec in a solution of trypsin inhibitor. Tissues were then dissociated mechanically in optiMEM-1 (Gibco) supplemented with glucose. Neurons in suspension (5 millions) were electroporated with Amaxa Nucleofector kit for rat neurons (Lonza) according to the supplier’s manual. Neurons were plated on glass coverslips coated with poly-L-lysine (1 mg/ml). After 3 hours, medium was replaced with fresh Neurobasal medium supplemented with 2% B27, 2 mM glutamax, 1% penicillin/streptomycin, 10 µM forskolin and 100 µM IBMX (Sigma-Aldrich).

### Microchambers and Neurons Plating

Microchambers and neuronal plating were previously described [31], [32]. Briefly, silicon wafer obtained by photolithography was used to create the master with SU8 resin. The channels are 3 µm high, 5 µm wide and 450 µm long. Silicon elastomer with its curing agent was used (PDMS, Sylgard 184, Dow corning) to create the devices. The microfluidic devices were then coated with poly-D-lysine (0.5 mg/ml) and laminin (10 mg/ml) overnight at 4°C and then washed three times with neuronal medium. One million neurons were plated in the cell body/proximal compartment of the chamber.

### Videomicroscopy

The microscopes and the chambers were kept at 37°C. Images were recorded every 200 ms with a 100 X PlanApo N.A. 1.4 oil immersion objective. For the gene replacement studies on neuronal rat culture, images were collected in stream mode using a Micromax camera (Roper Scientific) set at 2×2 binning. For DmHTT dynamics, images were collected using a spinning disk (Yokogawa CSU-X1 head) and a EM-CCD camera (Evolve) set at 1×1 binning. Projections, animations and analyses were generated using ImageJ software (http://rsb.info.nih.gov/ij/, NIH, USA). Maximal projection was performed to identify the vesicles paths, which in our system corresponds to vesicle movements in axons. Kymographs and velocity analyses were generated with the KymoToolBox, a homemade plug-in previously described [31].

## Results

### *Drosophila* N-terminal Huntingtin Fragment Interacts with Dynein Intermediate Chain

The N-terminal part of mammalian HTT interacts with proteins belonging to or associating with the molecular motor complex: there is a direct interaction with Dynein Intermediate Chain (DIC) [23] and an indirect interaction, via the Huntingtin Associated Protein 1 (HAP1) [33], with the p150*^Glued^* dynactin subunit [33], [34] and with Kinesin light Chain (KLC) [35]. Ectopic expression of terminal fragments of HTT containing these interacting domains allows fast axonal transport of vesicles [22], [25], [36], [37]. Conversely, full length HTT constructs lacking either the HAP1 or the dynein interacting domains alter or impair vesicular transport [38]. These results suggest that N-terminal HTT is necessary and sufficient to support transport of vesicles.

**Figure 1:**
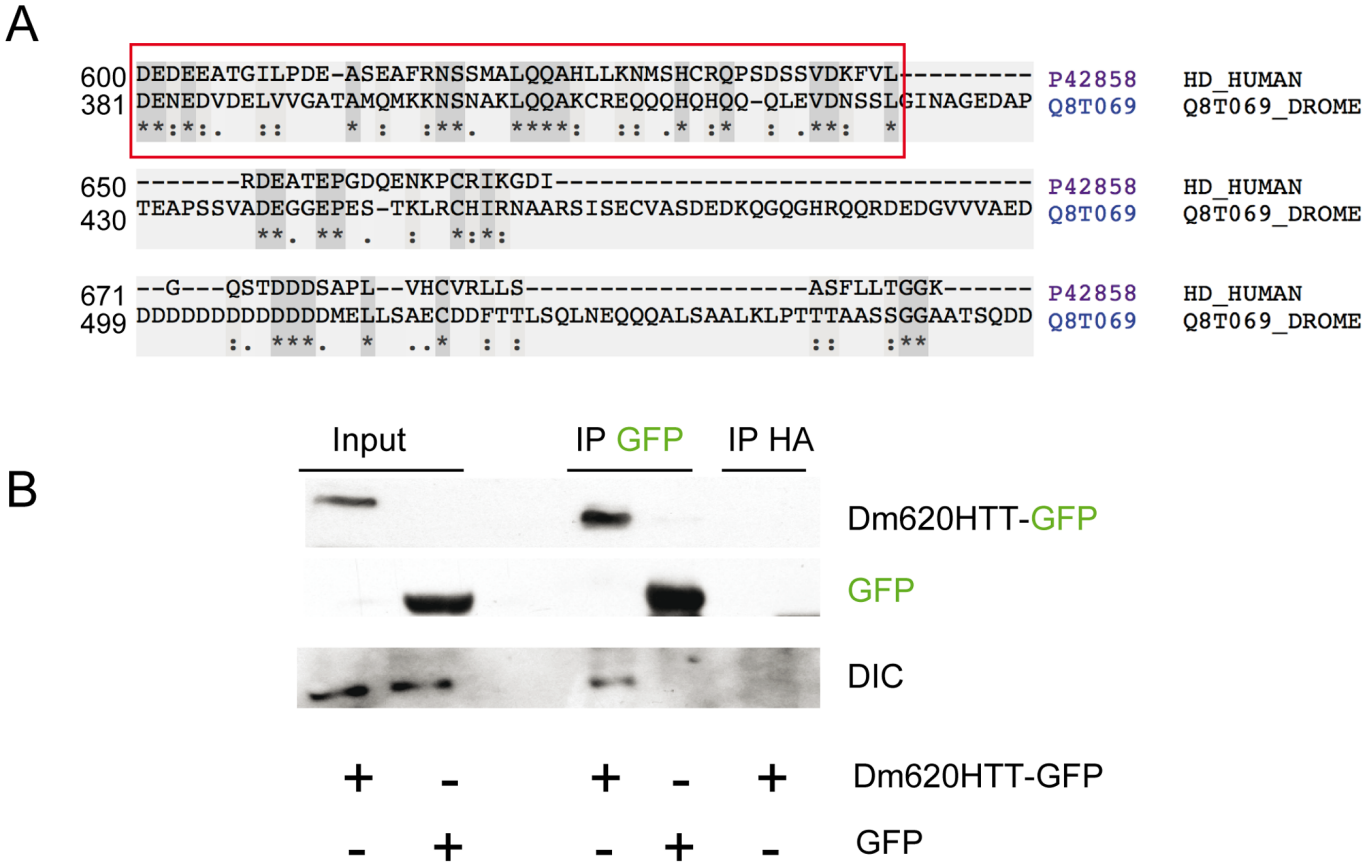
The dynein interacting domain of human HTT is conserved in *Drosophila melanogaster*. (A) Alignment of hHTT[600–698] and DmHTT[381–549]. The red box highlights the high homology region. (B) Western blot analyses of the co-immunoprecipitation of Dm620HTT-GFP with endogenous DIC on transfected HEK 293 cells: Dm620HTT-GFP but not control GFP co-precipitates DIC using an anti-GFP antibody. Anti-HA antibody was used as negative control and showed no Dm620HTT-GFP or DIC signal.

To test whether DmHTT functions in the transport of vesicles in flies, we first used an N-ter 620 amino-acid fragment of DmHTT. We previously showed that this fragment restores the spindle orientation defect of neuroblasts in HTT knock-out flies [29]. This function of HTT is associated with the recruitment of molecular motors at spindle poles and requires the motor proteins involved in vesicular trafficking. These findings suggested that this N-ter-620 amino-acid fragment of DmHTT may carry the domains allowing HTT binding to DIC. To investigate this possibility, we performed clustal analyses using various fragments of mammalian HTT and DmHTT. We limited the lengths of the mammalian HTT sequences analyzed on the basis of various findings: 1) Caviston and colleagues showed that human HTT fragment including amino-acids 600–1483 binds directly to dynein intermediate chain (DIC) and that a GST-HTT [536–698] fragment co-elutes with DIC of mouse brain cytosol [23]; this indicates that the minimal relevant region is [600–698]; 2) that partial deletion of this region from the full-length HTTΔ[633–672] abolished HTT transport function [38]. Clustal analyses showed that human HTT [600–698] partially aligns with the first 620 amino acids of DmHTT. In particular, the human HTT[600–649] and DmHTT[380–430] amino-acid sequences share an identity of 30% and a similarity of 62% (Fig. 1A).

We next investigated whether the N-Terminal 620 amino acid fragment of DmHTT interacts with DIC. HEK 293 cells were transfected either with the Dm620HTT-GFP or GFP constructs, and anti-GFP and control anti-HA antibodies were used for immunoprecipitation experiments. GFP and DmHTT-GFP were efficiently immunoprecipitated. DIC was only co-immunoprecipitated with Dm620HTT-GFP. Thus, fly HTT, like human HTT, interacts with human DIC (Fig. 1B).

### N-terminal Fragment of *Drosophila* Huntingtin Associates with Mammalian Vesicles

To investigate whether DmHTT acts in axonal transport of vesicles, we transfected HEK 293 cells with a cytosolic control protein (GFP) or with Dm620HTT-GFP and determined the distribution of the proteins in subcellular fractions.

**Figure 2:**
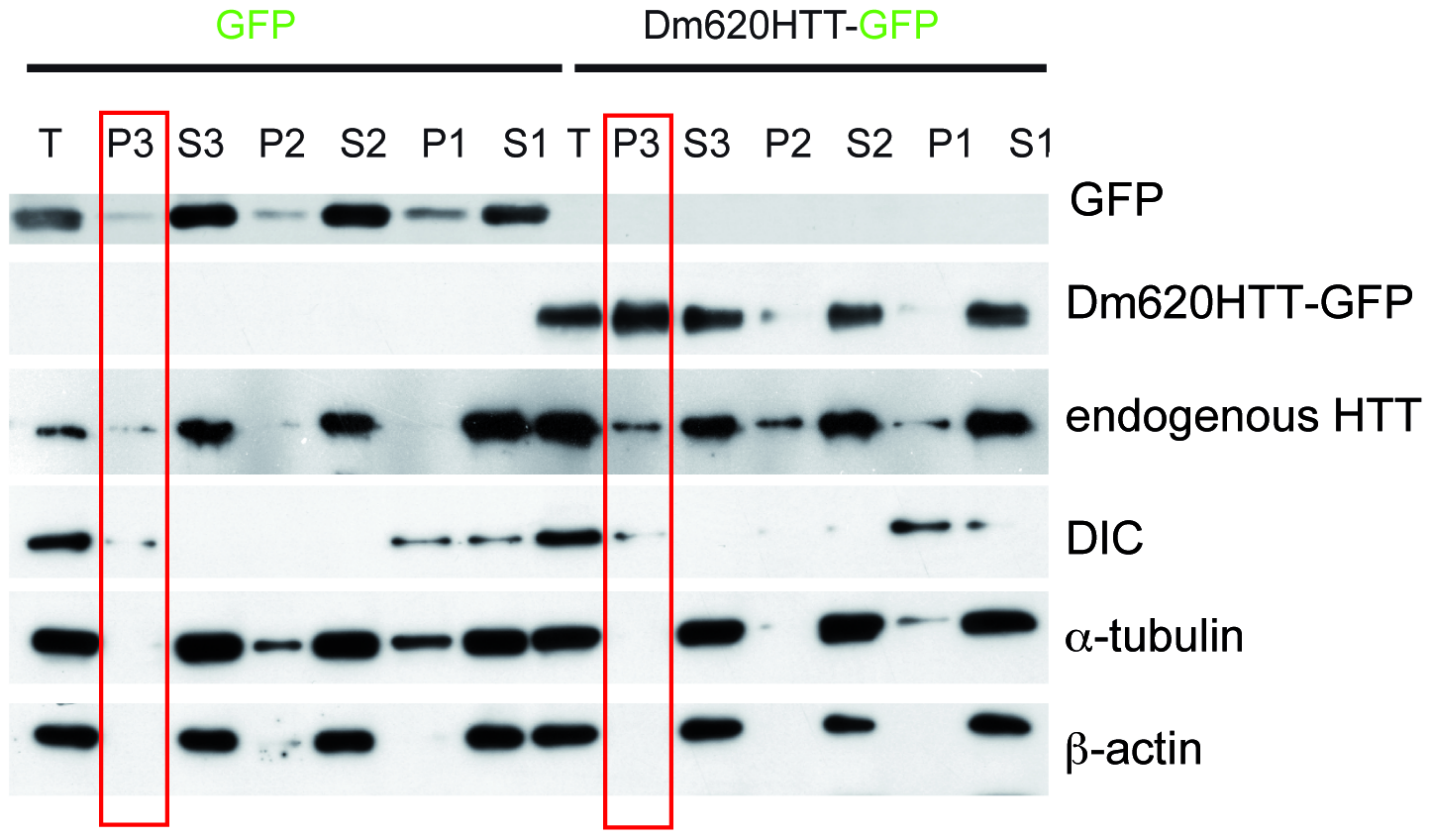
*Drosophila* Huntingtin N-terminal fragment associates to small vesicles. HEK 293 were transfected with GFP or Dm620HTT-GFP and cells were subjected to subcellular fractionation (T: total, P3: vesicles, S3: cytosol, P2: larges membranes, S2: cytosol plus light membranes, P1: nuclei and S1: post-nuclear supernatant) followed by Western blotting analyses to reveal the distribution of the different proteins (GFP, Dm620HTT-GFP, endogenous HTT, DIC, α-tubulin and β-actin). Immunoblot shows that GFP is mostly in the soluble fraction (S3) whereas Dm620HTT-GFP is enriched in the vesicles fraction (P3).

Western blotting analyses indicated that cytosolic proteins (α-tubulin and β actin) were abundant in the S3 fraction (the cytosol), and were scarce in the small membrane fraction P3 (vesicles and small membranes). As expected, endogenous mammalian HTT and DIC were present in both S3 and P3 fractions, confirming that a significant proportion of these proteins were associated with vesicles. Transfected GFP was mostly found in the S3 fraction and thus displayed a characteristic cytosolic profile. By contrast, Dm620HTT-GFP was mostly in the vesicular fraction, P3 (Fig. 2).

Thus, DmHTT associated with vesicles in mammalian cells, consistent with a putative function of fly HTT in dynein-dependent transport of vesicles along microtubules.

### *Drosophila* N-terminal Huntingtin Colocalizes with BDNF Vesicles in Axons

**Figure 3:**
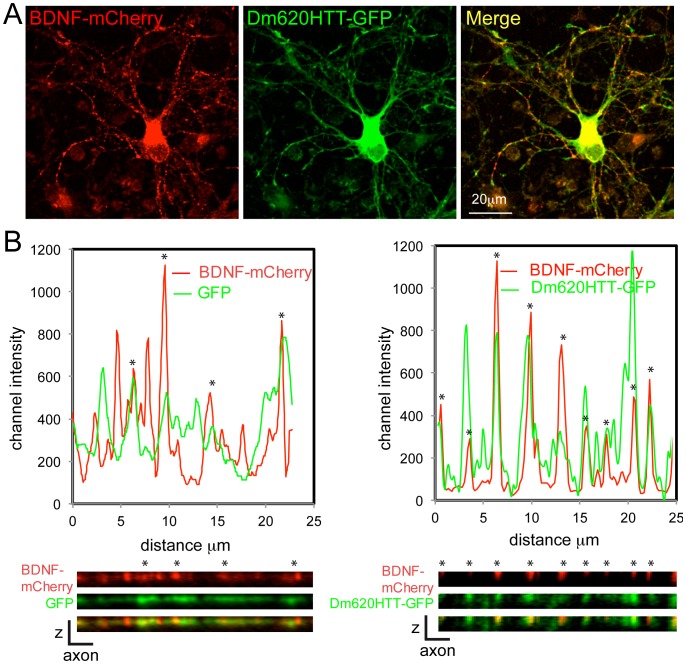
*Drosophila* Huntingtin N-terminal fragment colocalizes with neuronal BDNF vesicles. Rat cortical neurons were electroporated to express BDNF-mCherry and GFP or Dm620HTT-GFP, fixed and immunostained. Fluorescence was analyzed by confocal microscopy. (A) Confocal images showing the localization of both BDNF-mCherry and Dm620HTT-GFP in double-transfected neurons. (B) Resliced z-stack along an axon with channel intensity values (top) and immunostaining (bottom) with GFP channel (green) showing the localization of GFP (left) or Dm620HTT-GFP (right) and mCherry channel (red) showing the localization of BDNF-mCherry-containing vesicles. Whereas GFP intensity is diffuse and does not correlate with BDNF, Dm620HTT-GFP shows a punctate signal that partially co-localizes with BDNF. Stars show when the two peaks of intensity co-distribute in the line-scan and co-localize in the z-resliced images.

Mammalian HTT is involved in the vesicular transport of several types of cargos, including *Brain-Derived Neurotrophic Factor* (BDNF) [22], [25], [39]. Cortical neurons produce BDNF that is transported along the axons to the striatum where it is released [40]. The binding of BDNF to striatal postsynaptic TrkB receptors mediates survival of the striatal neurons. We tested whether DmHTT associates with BDNF vesicles by transfecting cortical rat neurons with Dm620HTT-GFP and BDNF-mCherry constructs. As expected, BDNF-mCherry showed a typical punctate staining in axons and dendrites, characteristic of vesicles (Fig. 3A). To assess possible co-localization of the two proteins, we re-sliced the stacks of images along neurites and analyzed their distribution intensities (Fig. 3B). BDNF-mCherry staining displayed characteristic vesicular pattern as shown by the presence of punctae within axons (Fig. 3B) and of marked peaks of intensity (Fig. 3B). In contrast, control GFP staining was more diffuse (Fig. 3B). Line-scan analysis revealed that the GFP channel fluctuations did not coincide with BDNF peak intensities. In contrast, the Dm620HTT-GFP signal was punctate and co-localized strongly with BDNF vesicles (Fig. 3B). Quantification of the line-scan further indicated that a significant percentage of BDNF vesicles (69.1% +/−2.5%, n = 343) were also positive for Dm620HTT-GFP. The number of peaks of DmHTT was greater than the number of BDNF vesicles, suggesting that, consistent with the wider role of mammalian HTT in transport, DmHTT was involved in the transport of cargo other than BDNF. These findings further implicate DmHTT in the axonal transport of vesicles.

### *Drosophila* Huntingtin Associates with Microtubules

**Figure 4:**
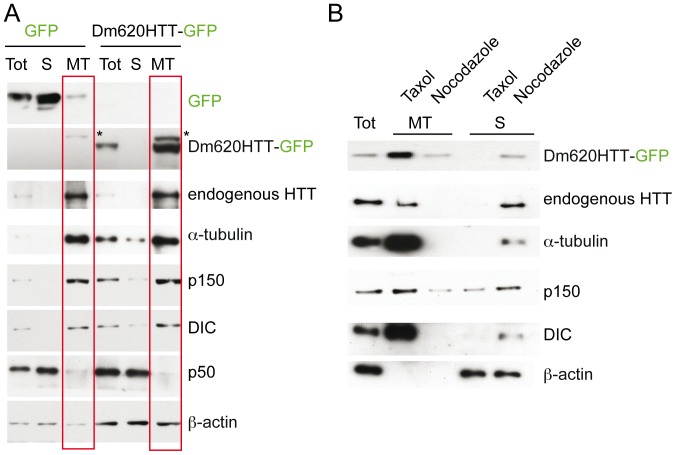
*Drosophila* Huntingtin N-terminal fragment associates with microtubules. HEK 293 were transfected with GFP or Dm620HTT-GFP and cell extracts were subjected to a microtubule depolymerization – repolymerization assay to pellet microtubules and the microtubule-associated proteins which were analyzed by Western blotting. (A) Whereas GFP does not pellet with the microtubules fraction (MT) but is soluble (S), Dm620HTT-GFP co-sediments with microtubules. (B) MT binding assay in presence of Taxol or Nocodazole. Dm620HTT-GFP is not pelleted in Nocodazole-treated cells when microtubules are depolymerized.

We next investigated whether DmHTT, like its mammalian homologue, associates with microtubules. We performed a microtubule depolymerization-repolymerization assay with transfected HEK 293 cells to analyze whether Dm620HTT-GFP co-sedimented with microtubules (MT) (Fig. 4A). We found α-tubulin to be enriched in the MT fraction and massively depleted from the soluble fraction (S) indicating that the assay efficiently purified MT-associated proteins. β-actin, the p50 dynamitin subunit of dynactin and GFP, that have no MT binding motifs, were found mostly in the soluble fraction (S). By contrast, dynein (DIC) and dynactin (p150*^Glued^*) were highly enriched in the MT fraction. Similarly, endogenous HTT and ectopically expressed Dm620HTT-GFP were recruited to microtubules. To investigate whether Dm620HTT-GFP was non-specifically associated with the MT fraction, we treated cell extracts with nocodazole to depolymerize microtubules. In these conditions, Dm620HTT-GFP was not precipitated, suggesting a specific association of DmHTT with MTs (Fig. 4B).

These various results in mammalian cells show that the N-terminal part of *Drosophila* HTT interacts with dynein, associates with the vesicular fraction, co-localizes with BDNF-containing vesicles in axons and associates with microtubules. Therefore, DmHTT, like HTT, appears to function in MT-based transport of vesicles.

### *Drosophila* Huntingtin Displays Fast Axonal Transport Properties and is Co-transported with BDNF Vesicles

**Figure 5:**
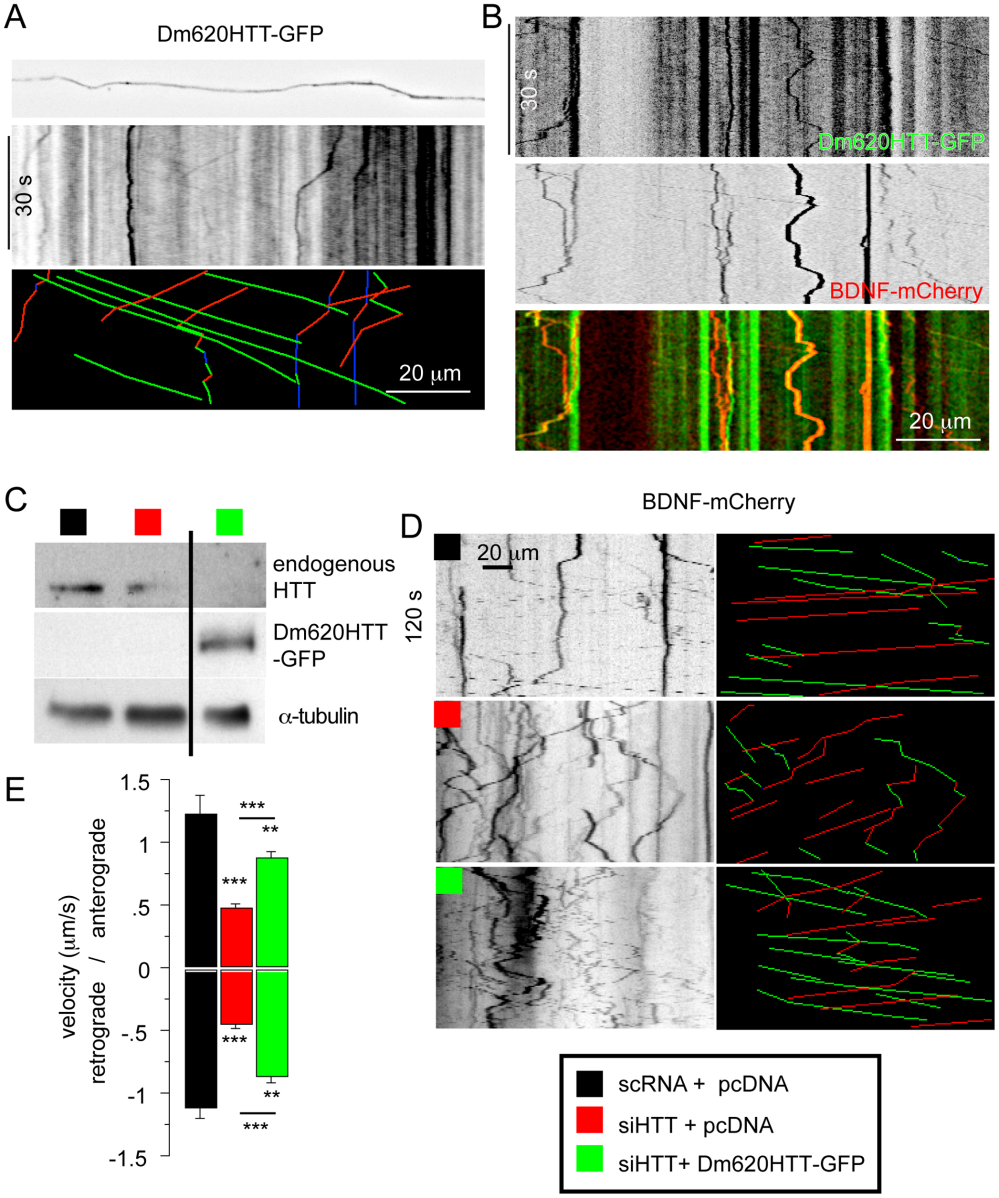
*Drosophila* Huntingtin is dynamic and rescues loss of mammalian huntingtin in axonal transport. (A) Dm620HTT-GFP is dynamic. Image within a microchannel, of an axon from a rat cortical neuron expressing Dm620HTT-GFP. The generated kymograph below shows the vesicular trajectories and the paths that were analyzed (green for anterograde moving vesicles, red for retrograde moving vesicles and blue for stationary vesicles). (B) Two-colors kymograph obtained from neurons co-transfected with BDNF-mCherry and Dm620HTT-GFP shows co-transport of DmHTT and BDNF-mCherry in axons. (C) Western blotting shows silencing of endogenous rat *Htt* gene and its replacement by fly *htt* in cortical neurons. (D) The generated kymograph shows the vesicular trajectory in the three conditions and the paths that were analyzed. (E) The mean velocity of anterograde and retrograde moving vesicles was calculated. (Mean+S.E.M, p* <0.05, p**<0.01 and p***<0.001).

We then investigated whether DmHTT could have a dynamic behavior in axons. We used a homemade microfluidic device derived from a previously described microfluidic culture platform to analyze transport in axons [31], [32], [41]. The device allows central nervous system axons to grow into fluidically isolated microchannels. Cortical neurons were electroporated with Dm620HTT-GFP construct and plated in the proximal compartment of the microfluidic chamber. Three to five days after plating, axons reached the distal chamber that is 450 µm away from the proximal compartment that contains the cell bodies and most of the dendrites [30], [39]. To analyze whether DmHTT displays fast axonal transport, we used spinning-disk confocal videomicroscopy to record the movement of GFP particles in the axons of cortical neurons expressing Dm620HTT-GFP. We observed diffuse fluorescence along the axons with some spots of greater fluorescence intensity, similar to the pattern observed in fixed and immunostained axons (Fig. 5A and Fig. 3B). Analyses of the movies and the associated kymographs revealed rapid anterograde and retrograde movements (Fig. 5A and Movie S1). The velocities of these movements were determined: Dm620HTT moved with a mean anterograde velocity of 2.7±0.2 µm/s (n = 34) and a mean retrograde velocity of 1.3±0.2 µm/s (n = 13). These velocities are typical of fast axonal transport. Having demonstrated that DmHTT is dynamic (Fig. 5A), we assessed whether DmHTT was co-transported with BDNF-containing vesicles. We electroporated embryonic rat cortical neurons with Dm620HTT-GFP and a construct encoding BDNF-mCherry. BDNF-mCherry is transported within axons with dynamics characteristic of fast axonal transport [31], [39], [42]. Analyses of BDNF vesicle dynamics revealed fast and highly processive movements (Fig. 5B) at velocities similar to those previously reported [31], [39], [42]. Two-color analysis of the dynamic behavior of Dm620HTT-GFP and BDNF-mCherry within an axon revealed the trafficking of both proteins (Movie S2). Kymograph analysis of the dynamics of the two proteins indicated that they were co-transported (Fig. 5B). We conclude that *Drosophila* HTT is dynamic and is associated with BDNF vesicles during their transport within axons.

### *Drosophila* Huntingtin Partially Rescues Loss of Mammalian HTT in Fast Axonal Transport

We then tested if endogenous mammalian HTT could be replaced by *Drosophila* HTT. We silenced rat HTT by treatment with a siRNA that was previously validated and does not have any off-target effects [25], [31]. This siRNA effectively decreased levels of endogenous HTT (Fig. 5C). We analyzed BDNF dynamics by videomicroscopy and observed, as previously demonstrated [22], [25], [36], that the siRNA caused a marked decrease in both anterograde and retrograde transport of BDNF (see the characteristic kymographs with colored trajectories and graphs of mean velocities, Fig. 5D and 5E). Next, we expressed the Dm620HTT-GFP construct in the rat HTT-silenced neurons; this construct is insensitive to the rat siHTT RNA (Fig. 5C). Videomicroscopy analyses of BDNF transport in these samples revealed a significant recovery of BDNF transport in both anterograde and retrograde (see kymograph analysis and BDNF velocities, Fig. 5D and 5E). Although the recovery to control values was not complete, this experiment demonstrates that *Drosophila* HTT facilitates axonal transport and partially restores mammalian HTT function.

### *Drosophila melanogaster* Huntingtin is Present in Fly Brains

**Figure 6:**
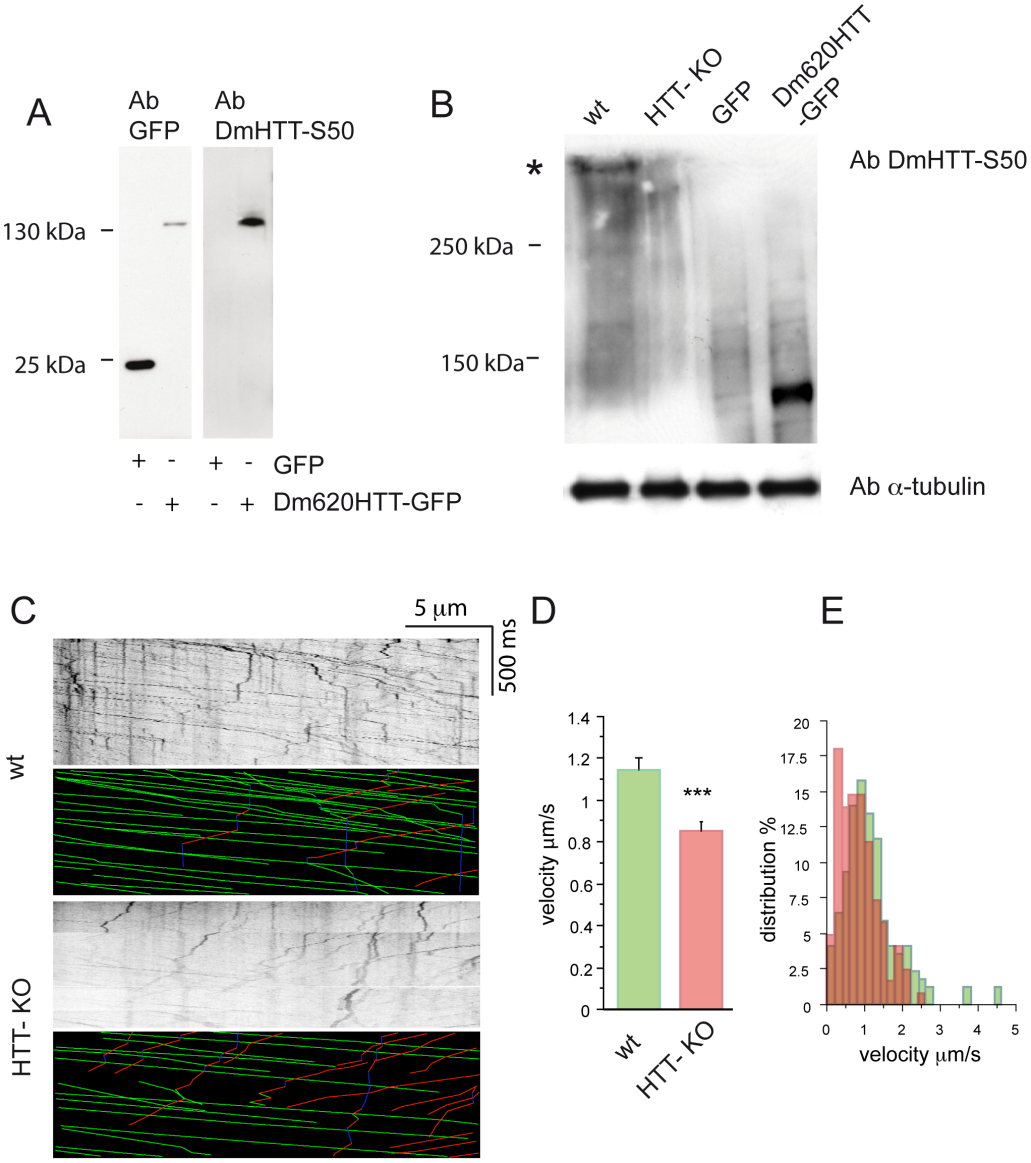
Fast axonal transport is impaired in *Drosophila* larvae knock out for HTT. (A) Anti DmHTT-S50 antibody recognizes Dm620HTT-GFP but not GFP when expressed in HEK 293 cells. (B) DmHTT-S50 recognizes a band of about 400 kDa in *Drosophila* L3 larvae brain extracts (see star). This band is absent in HTT-KO fly extracts. (C) Typical kymographs of wild-type and HTT-KO larvae with the analyzed trajectories for synaptotagmin-GFP vesicles. (D) Quantification of the mean velocity of axonal synaptotagmin-GFP vesicles in wild-type and HTT-KO larvae. (E) Distribution of synaptotagmin-GFP vesicles velocities shows a reduced number of fast-moving vesicles in HTT-KO larvae compared with wild-type flies.

Investigations of HTT function in flies have been impeded by the lack of antibodies recognizing the endogenous protein. The expression of the *HTT* gene in flies has been investigated by *in situ* hybridization: expression is ubiquitous but weak during development [26]. To analyze whether the HTT protein is produced in *Drosophila* brains, we raised antibodies, in rabbit, against two antigenic regions of the *D. melanogaster* protein sequence. The polyclonal antibody obtained recognized Dm620HTT-GFP but not GFP produced in HEK 293 cells (Fig. 6A). We tested whether this DmHTT antibody recognizes endogenous fly HTT, by testing third instar larval brains from wild-type and HTT-KO flies [26]. The antibody recognized the endogenous DmHTT, although the signal intensity was weaker than that for ectopically expressed Dm620HTT. Nevertheless, the antibody gave a specific band corresponding to the predicted size of DmHTT of 400 kDa (Fig. 6B); importantly, this band was absent from HTT-KO fly brain samples. DmHTT is therefore expressed in the brain of third instar larvae.

### Fast Axonal Transport is Impaired in *Drosophila* Huntingtin Knockout Mutants

To confirm that DmHTT is involved in axonal transport, we crossed *ok6-Gal4:UAS-Syt-GFP* flies, expressing the vesicular synaptic marker synaptotagmin-GFP in motoneurons, with DmHTT-KO flies [26]. Larvae at stage L3 obtained from these crosses were collected and placed between two coverslips. The dynamics of synaptotagmin vesicles were recorded by confocal videomicroscopy (Movie S3 and 4) [28], [31], [43], and kymographs were generated from the data collected (Fig. 6C). They revealed lower mean velocity of synaptotagmin in DmHTT-ko flies than in wild-type flies (Fig. 6D). Analysis of the velocity distribution revealed that there were fewer fast-moving vesicles in the HTT-KO than wild-type flies (Fig. 6E). These results demonstrate that DmHTT promotes axonal transport in flies and thus that this function is conserved between mammals and flies.

## Discussion

Almost two decades have passed since the identification of the CAG/polyglutamine expansion in the HTT gene/protein which leads to Huntington’s disease [3]. Despite intense research, HD remains incurable and has a devastating impact on patients’ lives. Various studies describe HTT as a scaffold or platform protein interacting with several different protein complexes that are involved in diverse cellular functions [14], [16]–[18], [20]. For example, HTT associates with transcription factors to regulate the expression of genes that, in turn, regulate neuronal survival [44]–[46]. HTT also regulates endo-exocytosis by interacting with components of the endo-exocytic and recycling machinery [47], [48]. As a result of HTT being part of these various complexes, it acts as a pro-survival and anti-apoptotic factor, regulates synaptic transmission and brain homeostasis, and is also involved in the control of cell maturation and differentiation [16], [18], [29], [49].

HTT also serves as a platform for the molecular motor complexes, dynein and kinesin [25], [50]. The interactions between HTT and these complexes have been extensively described [33]–[35]. Silencing or reducing HTT activity affects the capacity of the dynein/dynactin complex to function properly in various models: reducing wild-type HTT levels reduces axonal transport [22], [23], [25]; and it affects spindle orientation in neuronal progenitors, a mechanism that depends on the dynein/dynactin complex [29]. It also reduces ciliogenesis by altering the MT and dynein-dependent trafficking of cilia and basal body components to the base of the cilia [49]. Thus, the absence of HTT impairs these processes and as a consequence cells, especially neurons, accumulate several, albeit mild, cellular dysfunctions that lead to neurodegeneration as observed in HD [51]. The polyglutamine expansion also interferes with these functions. The polyQ expansion in HTT leads to defects in axonal transport [22] that are of similar severity as those associated with the loss of function phenotype induced by HTT silencing. Importantly, this effect is similar in ST*Hdh* ^Q109/+^ and ST*Hdh* ^Q109/Q109^ cells generated from heterozygotes and homozygotes knock-in mice [22], [52] and therefore appear to result from a dominant negative effect of the mutant protein over the wild-type protein. In addition to this mechanism, polyQ HTT expression in cells or mice also affects axonal transport due to the formation of neuritic aggregates that physically perturb intracellular trafficking [7], [24], [53]. The cellular phenotype (reduced intracellular transport of organelles and of protein complexes) observed in HD neurons may therefore result from the gain of new toxic functions as well as the loss of normal function(s).

*Drosophila melanogaster* has been widely used in HD research particularly for the overexpression of short N-ter fragments or full-length human HTT proteins with the polyQ expansion [4]–[7] this approach being based on a pure gain of function paradigm. The recent development of DmHTT knock-out flies facilitates the analysis of HTT functions in flies [26]. HTT inactivation in flies does not lead to the early embryonic lethality described in mice [54]. This difference is presumably the result of the essential role of mammalian HTT in extraembryonic tissues [51]. Therefore, progressive defects in HTT-KO flies may be analogous to the effects of inactivation of HTT in adult mice [19]. The phenotype of flies expressing the exon1 of HTT with 93Q worsens in the fly HTT-KO background, consistent with wild-type HTT having a role in adult flies [26]. This observation is very similar to the finding that the HD phenotype in mouse is modified by levels of wild-type HTT in heterozygotes [55]. The absence of HTT in flies may therefore result in mild dysfunctions similar to those described in mammalian models. Our findings that *Drosophila* HTT regulates axonal transport is in agreement with the previous study demonstrating that loss of HTT induces a spindle orientation defect of neuronal progenitors both in mammalian brain and in fly neuroblasts; spindle orientation depends on appropriate functioning of the dynein/dynactin complex [29]. The findings that HTT-KO flies recapitulate subtle phenotypes observed in mammals confirm that *Drosophila melanogaster* is a highly relevant model for studying HTT function and dysfunction in the context of HD.

## Supporting Information

Movie S1 **Movie shows Dm620HTT-GFP dynamics in rat axons located in the distal part of a microchannel.** The right part corresponds to the tracked vesicles within the channel. Green dots are for anterograde movements, red for retrograde movements and blue for stationary vesicles. Arrows indicate the position of vesicles within the axon.
(MOV)

Movie S2 **Movie shows from left to the right the dynamic behaviour in the distal part of a microchannel of the following proteins: Dm620HTT-GFP, BDNF-mCherry and overlay.** Arrows indicate the position of vesicles within the axon.
(MOV)

Movie S3 **The top movie shows the dynamic behaviour of synaptotagmin-GFP vesicles in motoneuron axons from control larvae and the bottom movie shows the vesicles that were subsequently tracked.** Green dots are for anterograde movements, red for retrograde movements and blue for stationary vesicles.
(MOV)

Movie S4 **The top movie shows the dynamic behaviour of synaptotagmin-GFP vesicles in motoneuron axons from DmHTT-KO flies and the bottom movie shows the vesicles that were tracked.** Green dots are for anterograde movements, red for retrograde movements and blue for stationary vesicles.
(MOV)
